# GFOGER Peptide Modifies the Protein Content of Extracellular Vesicles and Inhibits Vascular Calcification

**DOI:** 10.3389/fcell.2020.589761

**Published:** 2020-11-30

**Authors:** Ali Mansour, Walaa Darwiche, Linda Yaker, Sophie Da Nascimento, Cathy Gomila, Claire Rossi, Vincent Jung, Pascal Sonnet, Saïd Kamel, Ida Chiara Guerrera, Agnès Boullier, Jérôme Ausseil

**Affiliations:** ^1^MP3CV-UR7517, CURS-Université de Picardie Jules Verne, Amiens, France; ^2^AGIR, UR4294, UFR de Pharmacie, Université de Picardie Jules Verne, Amiens, France; ^3^Alliance Sorbonne Université, Université de Technologie de Compiègne, UMR7025 CNRS Enzyme and Cell Engineering Laboratory, Compiègne, France; ^4^Plateforme protéomique Necker, Faculté de Médecine Paris Descartes, Université de Paris – Structure Fédérative de Recherche Necker, INSERM US24/CNRS UMS3633, Paris, France; ^5^Laboratoire de Biochimie, Centre Hospitalier Universitaire d’ Amiens, Amiens, France; ^6^Centre de Physiopathologie Toulouse Purpan, INSERM UMR1043 – CNRS UMR5282 – Université Toulouse III, Toulouse, France; ^7^CHU Toulouse – Institut Fédératif de Biologie, Laboratoire de Biochimie, Toulouse, France

**Keywords:** vascular calcification, type I collagen, extracellular vesicle, oligogalacturonic acid, GFOGER sequence, osteogenic switch

## Abstract

**Objective:**

Vascular calcification (VC) is an active process during which vascular smooth muscle cells (VSMCs) undergo an osteogenic switch and release extracellular vesicles (EVs). In turn, the EVs serve as calcification foci via interaction with type 1 collagen (COL1). We recently showed that a specific, six-amino-acid repeat (GFOGER) in the sequence of COL1 was involved in the latter’s interaction with integrins expressed on EVs. Our main objective was to test the GFOGER ability to inhibit VC.

**Approach:**

We synthesized the GFOGER peptide and tested its ability to inhibit the inorganic phosphate (Pi)-induced calcification of VSMCs and aortic rings. Using mass spectrometry, we studied GFOGER’s effect on the protein composition of EVs released from Pi-treated VSMCs.

**Results:**

Calcification of mouse VSMCs (MOVAS-1 cells), primary human VSMCs, and rat aortic rings was lower in the presence of GFOGER than with Pi alone (with relative decreases of 66, 58, and 91%, respectively; *p* < 0.001 for all) (no effect was observed with the scramble peptide GOERFG). A comparative proteomic analysis of EVs released from MOVAS-1 cells in the presence or absence of Pi highlighted significant differences in EVs’ protein content. Interestingly, the expression of some of the EVs’ proteins involved in the calcification process (such as osteogenic markers, TANK-binding kinase 1, and casein kinase II) was diminished in the presence of GFOGER peptide (data are available via ProteomeXchange with identifier PXD018169^∗^). The decrease of osteogenic marker expression observed in the presence of GFOGER was confirmed by q-RT-PCR analysis.

**Conclusion:**

GFOGER peptide reduces vascular calcification by modifying the protein content of the subsequently released EVs, in particular by decreasing osteogenicswitching in VSMCs.

## Highlights

– Incubation with synthetic GFOGER peptide was associated with significantly lower Pi-induced calcification in vascular smooth muscle cells and aortic rings.
– GFOGER peptide reduces vascular calcification by modifying the protein content of VSMCs-derived EVs.
– GFOGER peptide decreases the osteogenic switching in VSMCs.

## Introduction

Vascular calcification (VC) is a life-threatening cardiovascular complication associated with a high mortality rate—especially in a context of diabetes, atherosclerosis, or chronic kidney disease (CKD) (Adeney et al., 2009; Wu et al., 2013; Russo et al., 2014; Schlieper et al., 2016). This calcification is characterized by the deposition of hydroxyapatite crystals in the intimal and medial layers of coronary and/or peripheral arteries (i.e., intimal and medial calcification), leading to cardiovascular consequences such as congestive heart failure, left ventricular hypertrophy, aortic stenosis, and hypertension (Demer and Tintut, 2008; Nicoll and Henein, 2014; Chow and Rabkin, 2015; Durham et al., 2018). Over the last 10 years, extensive research has improved our understanding of the pathophysiology of VC. It is now well established that VC is an active process, with features of bone physiology and metabolism (Demer and Tintut, 2008; Thompson and Towler, 2012; Voelkl et al., 2019). For example, the calcium and phosphate mineral imbalances in CKD lead to the reprogramming of vascular smooth muscle cells (VSMCs), which become osteoblast-like cells. This osteogenic switching is driven by the expression of several bone markers, such as Runt-related transcription factor 2 (Runx2), alkaline phosphatase (ALP), osteopontin (OPN), matrix-Gla protein (MGP), osteocalcin (OCN), type 1 collagen (COL1), and bone sialoproteins (Xiao et al., 2005; Speer Mei et al., 2009; Durham et al., 2018).

Recent research has shown that VSMCs secrete heterogeneous populations of extracellular vesicles (EVs) (New Sophie, 2013). Under physiological conditions, EVs contain high levels of calcification inhibitors, such as fetuin-A and MGP (Reynolds et al., 2004, 2005; Kapustin et al., 2011). Under pathological conditions, however, the secreted EVs acquire a procalcifying profile and thereby act as nucleating foci for hydroxyapatite crystallization and propagation (New Sophie, 2013; Kapustin et al., 2015). Electron microscopy-based studies have revealed that these EVs are embedded between the collagen and elastin fibrils of arterial walls—suggesting that calcification can be initiated by direct physical interaction with EVs (Chen et al., 2008; Kapustin et al., 2011).

We previously reported that a specific oligogalacturonic acid with a degree of polymerization of 8 (DP8) was able to inhibit VC by diminishing osteogenic marker expression, masking a consensus amino acid repeat found in COL1 (sequence: GFOGER), and thus preventing EVs from binding. Although the *in vivo* use of DP8 appears to be compromised (probably due to enzymatic digestion), our results suggested that the osteogenic switching of VSMCs and the prevention of EV–COL1 interactions were therapeutic targets for inhibiting VC (Hodroge et al., 2017). Because we have already shown that the triple-helical GFOGER consensus sequence forms a preferential binding site for EVs (Hodroge et al., 2017), we decided to chemically synthesize a GFOGER peptide and determine its ability to inhibit VC on VSMCs *in vitro* and in an *ex vivo* aortic ring model. We also sought to characterize the mechanism of VC inhibition by investigating EVs’ protein content in the presence of the GFOGER peptide.

## Materials and Methods

### Cell Culture, Molecular and Biochemical Reagents

Dulbecco’s modified Eagle’s minimal essential medium (DMEM 6546), fetal calf serum (FCS), and Dulbecco’s phosphate buffered saline solution were obtained from Eurobio (Paris, France). Exosome-free fetal bovine serum (FBS) and trypsin were obtained from Dominique Dutcher (Brumath, France) and PAN Biotech (Aidenbach, Germany), respectively. RNase-free water, the Maxima First Strand cDNA kit, and Power SYBR green PCR Master Mix were bought from ThermoFisher Scientific (Warrington, United Kingdom). All other chemicals and biologicals were purchased from Sigma–Aldrich (St. Louis, MO, United States).

### Peptide Synthesis

The GFOGER and GOERFG peptides were synthesized using a standard, automated, continuous-flow, solid-phase method with double coupling (Liberty1, CEM), as described previously (Pêcher et al., 2009; Hodroge et al., 2017). The completed peptides were cleaved from the resin and side-chain unprotected by treatment with the scavengers water/triisopropylsilane/trifluoroacetic acid (2.5:2.5:95 v/v/v) for 30 min under microwave radiation (Discover, temperature: 38°C, power: 20 W). The corresponding peptides were purified by reverse-phase HPLC using a Shimadzu preparative HPLC system on an RP-HPLC C12 column (Phenomenex C12 Jupiter proteo, 90 Å, 21.2 × 250 mm) with a mixture of aqueous 0.1% (v/v) trifluoroacetic acid (A) and 0.1% (v/v) trifluoroacetic acid in acetonitrile (B) as the mobile phase (flow rate of 15 ml/min) and employing UV detection at 220 nm. Characterization of the peptides were performed by mass spectrometry on a LC-HRMS, and analyses were performed on an ACQUITY UPLC H-Class system (Waters-Micromass, Manchester, United Kingdom) coupled with a SYNAPT G2-Si Q-TOF hybrid quadrupole time-of-flight instrument (Waters-Micromass), equipped with an electrospray (ESI) ionization source (Z-spray) and an additional sprayer for the reference compound (Lock Spray, Torrance, CA, United States) heated at 50°C. The source and dissolving temperatures were 120 and 450°C, respectively. Nitrogen was used as a drying and nebulizing gas at flow rates of 50 and 900 L/h, respectively. Typically, the capillary voltage was 3 kV, the sampling cone voltage was 40 V, and the source offset was 40 V. Lock mass corrections using [M + H]^+^, 120.0813 *m/z*, and 556.2771 *m/z* of a leucine-enkephalin solution (50 pg μl^–1^ in 50:50 acetonitrile/water + 0.1% formic acid) were applied for accurate mass measurements (elemental composition determination). The mass range was 50–2000 Da and spectra were recorded at 0.2 s/scan in the centroid mode at a resolution of 20,000 (FWHM) in the resolution mode. Data acquisition and processing were performed with MassLynx 4.1 software.

The predicted and observed high resolution masses for GFOGER [C_29_H_44_N_9_O_10_: (M + H)^+^] were 678.3211 and 678.3246, respectively (Hodroge et al., 2017) and GOERFG [C_29_H_43_N_9_O_10_: (M + H)^+^] were 678.3209 and 678.3211, respectively.

### Culture and Treatment of Cells and Aortic Rings

#### Murine Vascular Smooth Muscle Cell Culture

Murine aortic VSMCs (MOVAS-1 CRL-2797, ATCC, Manassas, VA, United States) were maintained in DMEM 6546 medium supplemented with 10% FCS, 100 IU/ml penicillin, 100 μg/ml streptomycin, 4 mM glutamine, and 200 μg/ml geneticin (G418) at 37°C in a humidified 5% CO_2_ atmosphere. The MOVAS-1 cells were first seeded in 48-well plates at a density of 4,500 cells/well. At 80% confluence, cells were treated in DMEM 6546 containing 1% FCS with serial dilutions of the GFOGER peptide (5, 25, 50, 100, 150, 250, and 500 μM) in the presence or absence of 4 mM inorganic phosphate (Pi) for 10 days. The medium was renewed twice a week.

#### Human Vascular Smooth Muscle Cell Culture

Aortic explants were obtained from patients having undergone various types of surgery at Amiens University Medical Center (Amiens, France). The study was approved by the *Comité de Protection des Personnes Nord-Ouest II* (Amiens, France; reference: 2009/19). The investigations were performed according to the principles outlined in the Declaration of Helsinki for the use of human tissues or subjects. Endothelium was removed from the aortic explants and the medial tissue was separated from the aortic segment. Next, small pieces (1–2 mm^2^) of the medial tissue were cultured in Petri dishes containing DMEM 6546 with 15% FBS, allowing cells to migrate out of the explant. At confluence, cells were harvested and cultured in T75 flasks. The VSMCs were then cultured in DMEM 6546 supplemented with 15% FBS, 100 IU/ml penicillin, 100 μg/ml streptomycin, and 4 mM GlutaMax at 37°C in a humidified 5% CO_2_ atmosphere. Primary human VSMCs from three different donors were seeded into 48-well plates at a density of 6,000 cells/well. When 80% confluence was achieved, the cells were treated with GFOGER (500 μM) in the presence or absence of 4 mM Pi in DMEM 6546 with 1% FBS for 14 days. The medium was renewed twice a week.

#### Preparation and Culture of Aortic Rings

Wild-type male Wistar rats were handled in accordance with French and European legislation (Directive 2010/63/EU), as previously described (Hodroge et al., 2017). After sacrifice, thoracic aortas were isolated and then directly placed in DMEM 6546 with 10% FCS (Baker et al., 2011). Aortic rings (3–5 mm) were incubated in DMEM 6546 supplemented with 10% FCS, 50 IU/ml penicillin, 50 μg/ml streptomycin, and 2 mM GlutaMax at 37°C in a humidified 5% CO_2_ atmosphere. In the treatment experiments (500 μM GFOGER peptide), aortic rings were incubated in DMEM 6546 with 10% FCS in the absence or presence of 4 mM Pi. After 10 days of culture (with renewal of the medium every 2 days), aortic rings were washed twice with Ca^2+^- and Mg^2+^-free PBS before measurement of the intracellular calcium content using the O-cresolphtalein (OCP) method (Sarkar and Chauhan, 1967).

### Biochemical Assays

#### WST-1 Cell Viability Assay

MOVAS-1 cells were seeded in 96-well plates at a density of 3,200 cells/well, cultured for 2 days (reaching 80% confluence), and then treated for 72 h with either medium alone, 4 mM Pi, 500 μM GFOGER or Pi + 500 μM GFOGER. One hundred microliters of diluted WST-1 reagent (1/10 in DMEM medium) was added per well. After a 1-h incubation at 37°C, optical densities (ODs) were spectrophotometrically measured at 450 nm.

#### Calcification Assays

##### Intracellular calcium content

The intracellular calcium content was assessed using the OCP assay as previously described (Sarkar and Chauhan, 1967). Furthermore, 50 μl of 0.1 N NaOH was added per well, to determine the cell protein content using Peterson’s method; the intracellular calcium concentration was normalized against this value (Peterson, 1977).

##### Alizarin Red staining

Aortic calcification was assessed using Alizarin Red staining (Issa et al., 2019). Briefly, aortas were embedded in OCT compound and then frozen in cold (−80°C) isopentane. The frozen aortas were then cut (using a Leica cryostat) to obtain thin aortic rings (thickness: 7 μm), which were placed on slides. For staining, a few drops of Alizarin Red solution (pH 4.1) were placed on the slides for 2 min at room temperature. After removal of the Alizarin Red solution, the slides were immersed successively in three different baths: acetone (100%, 1 min), acetone–toluene (1:1, 1 min), and toluene (100%, 5 min). Lastly, slides were dried at room temperature overnight. The Alizarin Red staining was visualized using an AxioImager D2 microscope (Zeiss, New York, NY, United States) and analyzed using HistoLab software (Stansfield, United Kingdom).

### Extracellular Vesicles’ Extraction

MOVAS-1 cells were seeded in 10-cm Petri plates (600,000 cells/Petri plate), cultured in DMEM 6546 medium supplemented with 10% FCS for 2 days and then treated with 1% exosome-free FBS (ThermoFisher Scientific, New York, NY, United States) for 10 days. EVs were extracted from culture supernatants by sequential centrifugations as described previously by Théry et al. (2006). Briefly, cells and dead cells were first removed by 10-min centrifugations at 300 × *g* and 2,000 × *g*. Then, the collected supernatant was centrifuged at 10,000 × *g* for 30 min to get rid of large cellular debris. After centrifugation, the supernatant was recovered and centrifuged at 100,000 × *g* for 70 min. The pellet was then resuspended in cold PBS and centrifuged at 100,000 × *g* for another 70 min. The obtained pellet which contains EVs was resuspended in PBS then immediately stored at -80°C for future analysis. The pellets’ protein concentrations were determined with a BCA Protein Assay Reagent Kit (ThermoFisher Scientific, Rockford, IL, United States).

### Mass Spectrometry Proteomic Analysis

#### Suspension Trapping

A suspension trapping S-Trap micro spin column (ProtiFI, Huntington, VI, United States) digestion was performed on 10 μg of exosome lysate, according to the manufacturer’s instructions. Briefly, 5% SDS and tris (2-carboxyethyl) phosphine hydrochloride were added to the samples and thus reducing and alkylating, respectively, giving a final protein concentration of 20 mM and a final chloroacetamide concentration of 40 mM. Next, 1.2% aqueous phosphoric acid was added to the final solution. A colloidal protein particulate was formed by the addition of six times the sample volume of S-Trap binding buffer (90% aqueous methanol, 100 mM triethylammonium bicarbonate, pH 7.1). The mixtures were added to S-Trap micro 1.7-ml columns and centrifuged at 4,000 × *g* for 30 s. The columns were washed four times with 150 μl S-Trap binding buffer and centrifuged at 4,000 × *g* for 30 s, with 180° rotation of the columns between washes. Samples were digested with 0.5 μg trypsin (Promega, Madison, WI, United States) at 47°C for 90 min. Peptides were eluted with 40 μl of 50 mM triethylammonium bicarbonate buffer (Sigma–Aldrich), followed by 40 μl of 0.2% aqueous formic acid and then 35 μl of 50% acetonitrile containing 0.2% formic acid. Lastly, the peptides were vacuum dried.

#### Protein Identification and Quantification Using Nano-Liquid Chromatography–Mass Spectrometry

Samples were resuspended in 20 μl of 10% acetonitrile/0.1% trifluoroacetic acid in HPLC-grade water. For each sample, three runs (injection volume: 1 μl) were performed on a nanoRSLC-Q Exactive PLUS system (RSLC Ultimate 3000; ThermoFisher Scientific, Waltham, MA, United States). Peptides were loaded on a micro pre-column (Acclaim PepMap 100 C18, cartridge, 300 μm i.d. × 5 mm, 5 μm) (ThermoFisher Scientific, Waltham MA, United States) and separated on a 50-cm reverse-phase liquid chromatographic column (0.075 mm ID, Acclaim PepMap 100, C18, 2 μm; ThermoFisher Scientific, Waltham MA, United States). The chromatography solvents were 0.1% formic acid in water (A) and 0.08% formic acid in 80% acetonitrile (B). The peptides were eluted from the column with the following gradient: 5–40% B (120 min) and 40–80% B (1 min). At 121 min, the gradient was held at 80% B for 5 min. At 127 min, the gradient returned to 5% B for 20 min, to re-equilibrate the column before the next injection. Two blanks were run between each series, and one blank was run between each sample to prevent sample carryover. Peptides eluted from the column were analyzed using data-dependent MS/MS (the top-10 acquisition method) and fragmented using higher-energy collisional dissociation. The instrument settings were as follows: resolution, 70,000 for MS scans and 17,500 for the data-dependent MS/MS scans (to increase the speed); MS AGC target, 3 × 10^6^ counts with a maximum injection time of 60 ms; MS/MS AGC target, 1 × 10^5^ counts with a maximum injection time of 120 ms; MS scan range, 400–2000 *m/z*; dynamic exclusion, 30 s.

#### Data Processing After the LC-MS/MS Acquisition

Raw MS data files were processed with MaxQuant software (version 1.5.3.30) and searched with the Andromeda search engine against the *Mus musculus* Uniprot KB/Swiss-Prot database (version 06/2016). To search for parent mass and fragment ions, the initial mass deviation was set to 4.5 and 20 ppm, respectively. The minimum peptide length was set to seven amino acids. Strict specificity for trypsin cleavage was required, allowing for up to two missed cleavage sites. Cysteine carbamidomethylation was set as a fixed modification, whereas methionine oxidation and N-terminal acetylation were set as variable modifications. The false discovery rate (FDR) was set to 1% for proteins and peptides. Scores were calculated using MaxQuant, as described previously (Cox and Mann, 2008). The reverse and common contaminant hits were removed from MaxQuant’s output. Proteins were quantified according to MaxQuant’s label-free algorithm, using label-free quantification intensities (Cox and Mann, 2008; Luber et al., 2010). Protein quantification was obtained using at least two peptides per protein.

Three independent isolations of EVs from MOVAS-1 cells treated (or not) with Pi were analyzed with Perseus software (version 1.6.2.3^1^). The label-free quantification data were log2-transformed, and proteins identified in the three replicates were retained for statistical testing. To simulate the distribution of low signal values, the missing values were imputed using a Gaussian distribution of random numbers with a standard deviation of 30% (relative to the SD of the measured values) and a 1.8-SD downshift of the mean. Four groups of samples were defined: non-treated (controls), Pi-treated, GFOGER-treated, and Pi- and GFOGER-treated. A *t*-test was used to compare treatment groups with the control (FDR: 0.05; S0: 0.1), and the data are represented as volcano plots. Lastly, the MS proteomics data were deposited with the ProteomeXchange Consortium via the PRIDE (Perez-Riverol et al., 2019) partner repository (dataset identifier: PXD018169).

### RNA Extraction and Quantitative Real-Time PCR

Total RNA was extracted from MOVAS-1 cells using a mixture of Trizol/CHCl_3_ (1/0.2; v/v). Total RNA was extracted from MOVAS-1 cells using TRIZOL reagent, separated into a distinct phase by the addition of chloroform, and then collected in a new tube. The RNA was precipitated with isopropanol and then washed with 75% ethanol to remove organic and protein contaminants. Lastly, dry RNA extracts were resuspended in 30 μl of RNase-free water. The RNA concentration was measured in a NanoVue Plus spectrophotometer (Holliston, MA, United States). cDNA was synthesized using a Maxima First Strand cDNA kit. For gene expression analysis, cDNA was amplified in a quantitative real-time (qRT) PCR thermocycler (BIO-RAD, Hercules, CA, United States) using Power SYBR Green PCR Master Mix and specific primers for the genes of interest (Table 1). The housekeeping gene *ARP0* was used to normalize gene expression levels.

**TABLE 1:** Forward and reverse primers used to quantify transcription of the genes of interest.

**Gene**	**Primer**	**Sequence**
*ARP0*	Forward	5′-TCCAGAGGCACCATTGAAATT-3′
	Reverse	5′-TCGCTGGCTCCCACCTT-3’
*Runx2*	Forward	5′-AGGCACAGACAGAAGCTTGATG-3′
	Reverse	5′-GCGATCAGAGAACAAACTAGGTT-3′
*OCN*	Forward	5′-GACCGCCTACAAACGCATCT-3′
	Reverse	5′-GGGCAGCACAGGTCCTEEATAGT-3′
*MGP*	Forward	5′-ATGAAGAGCCTGCTCCCTCT-3′
	Reverse	5′-ATATTTGGCTCCTCGGCGCT-3′
*TNAP*	Forward	5′-CTGCCACTGCCTACTTGTGT-3′
	Reverse	5′-GATGGATGTGACCTCATTGC-3′
*SMPD3*	Forward	5′-AGAAACCCGGTCCTCGTACT-3′
	Reverse	5′-CCTGACCAGTGCCATTCTTT-3′

### Statistical Analysis

Quantitative data were expressed as the mean ± SEM and were analyzed using GraphPad Prism software (version 7.0, San Diego, CA, United States). In a one-way ANOVA or a *t*-test, the threshold for statistical significance was set to *p* < 0.05.

## Results

Different concentrations of GFOGER peptide (5–500 μM) were tested on the MOVAS-1 cell line in the presence of 4 mM Pi, to determine the optimal concentration range for testing calcification inhibition. Only the concentrations of 250 and 500 μM inhibited calcification (data not shown).

### GFOGER Peptide Does Not Affect the Viability of MOVAS-1 Cells

**FIGURE 1:**
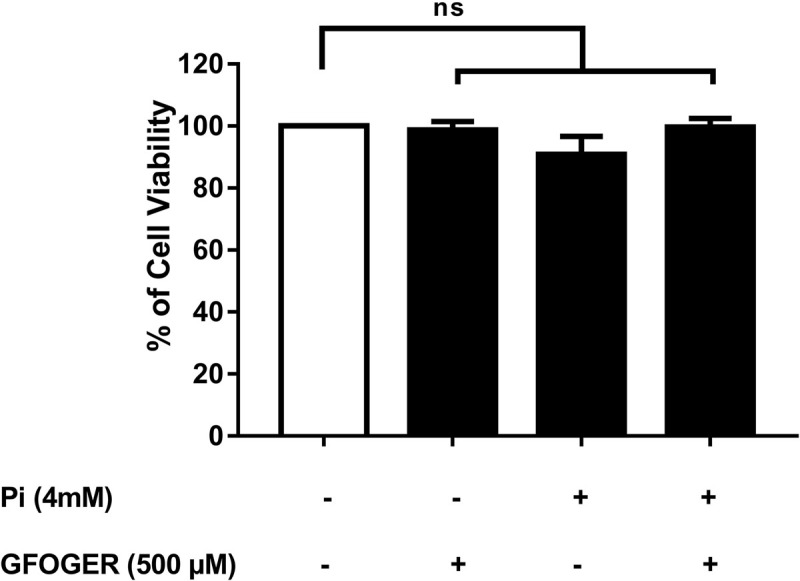
Effect of GFOGER peptide on MOVAS-1 cells’ viability. MOVAS-1 cells were incubated with 500 μM GFOGER peptide for 72 h in presence or absence of 4 mM Pi. Cell viability was measured using the WST-1 assay. Cell viability of untreated control cells was taken as 100%. Data are expressed as mean ± SEM of three independent experiments done in triplicate (*n* = 3). Parametric one-way ANOVA test.

Cell viability was assessed after a 72-h incubation in the presence or absence of the highest inhibitory concentration of GFOGER peptide (i.e., 500 μM). No cytotoxicity was observed—even when 4 mM Pi was added (vs. medium alone; Figure 1). We therefore decided to study both concentrations (250 and 500 μM GFOGER) in our subsequent experiments.

### GFOGER Peptide Inhibits the Pi-Induced Calcification on Smooth Muscle Cells

**FIGURE 2:**
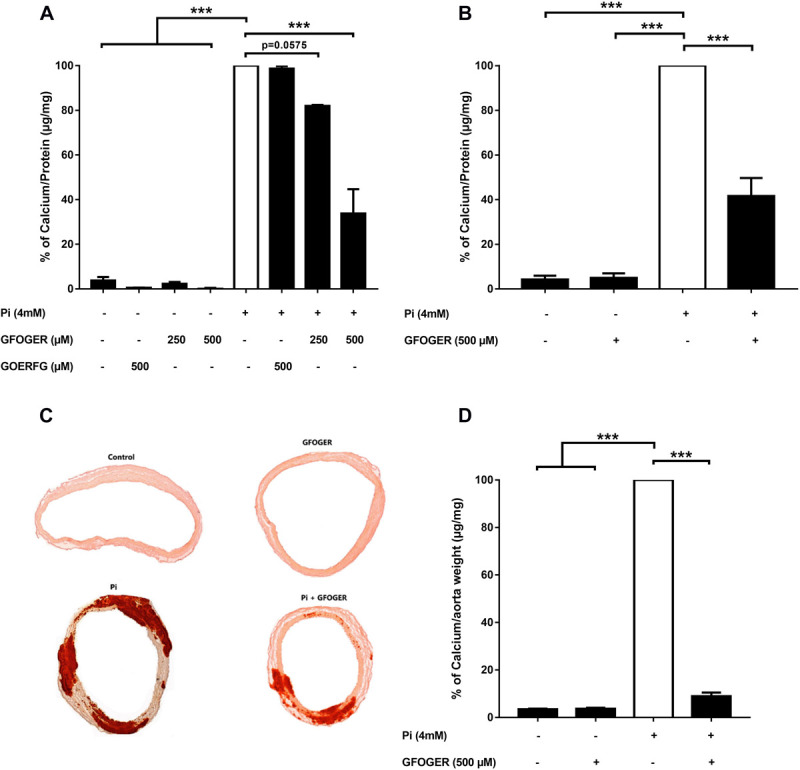
GFOGER peptide inhibits Pi-induced calcification: **(A,B)** in smooth muscle cells. Calcification in smooth muscle cells was induced by incubation with 4 mM Pi in absence or presence of GFOGER peptide or GOERFG peptide. **(A)** MOVAS cells were incubated with two concentrations of GFOGER peptide (250 and 500 μM) or with 500 μM of GOERFG peptide for 10 days. **(B)** Primary human vascular smooth muscle cells were incubated with 500 μM of GFOGER peptide for 14 days. Calcification was then measured using the OCP method. **(C,D)** In rat aortic rings. Rat aortic rings were incubated with 4 mM Pi in presence or absence of 500 μM GFOGER peptide for 10 days. **(C)** Images of one representative Alizarin Red staining experiment are shown. **(D)** Intracellular calcium content was quantified by OCP colorimetric method. Data are expressed as mean ± SEM of three independent experiments done in triplicate (*n* = 3). ***p < 0.001 vs. 4 mM Pi. Parametric one-way ANOVA test.

At concentrations of 250 and 500 μM, GFOGER peptide was associated with a lower level of Pi-induced calcification in MOVAS-1 cells; however, only the difference at 500 μM was statistically significant (^∗∗∗^*p* < 0.001 vs. 4 mM Pi; Figure 2A). In contrast, GOERFG peptide (used as scramble peptide) was not able to inhibit calcification and was equivalent to basic condition (Figure 2A). This result was confirmed on primary human VSMCs, in which Pi-induced calcification was significantly lower (by up to 58%) in the presence of 500 μM GFOGER peptide (^∗∗∗^*p* < 0.001 vs. 4 mM Pi; Figure 2B).

### GFOGER Peptide Inhibits the Calcification of Aortic Rings

We next investigated the GFOGER peptide’s effect in an *ex vivo* rat aortic ring model. Pi-induced mineralization of the aortic rings, as revealed by the Alizarin Red staining, was drastically lower in the presence of GFOGER compared with the Pi alone condition (Figure 2C). The calcium level in the aortic rings (according to an OCP assay) was significantly lower (by up to 91%) in the presence of 500 μM GFOGER peptide (^∗∗∗^*p* < 0.001 vs. 4 mM Pi, Figure 2D).

Taken as a whole, these results confirm that the GFOGER peptide specifically inhibits Pi-induced calcification.

### GFOGER Peptide Does Not Normalize SMPD3 Expression

**FIGURE 3:**
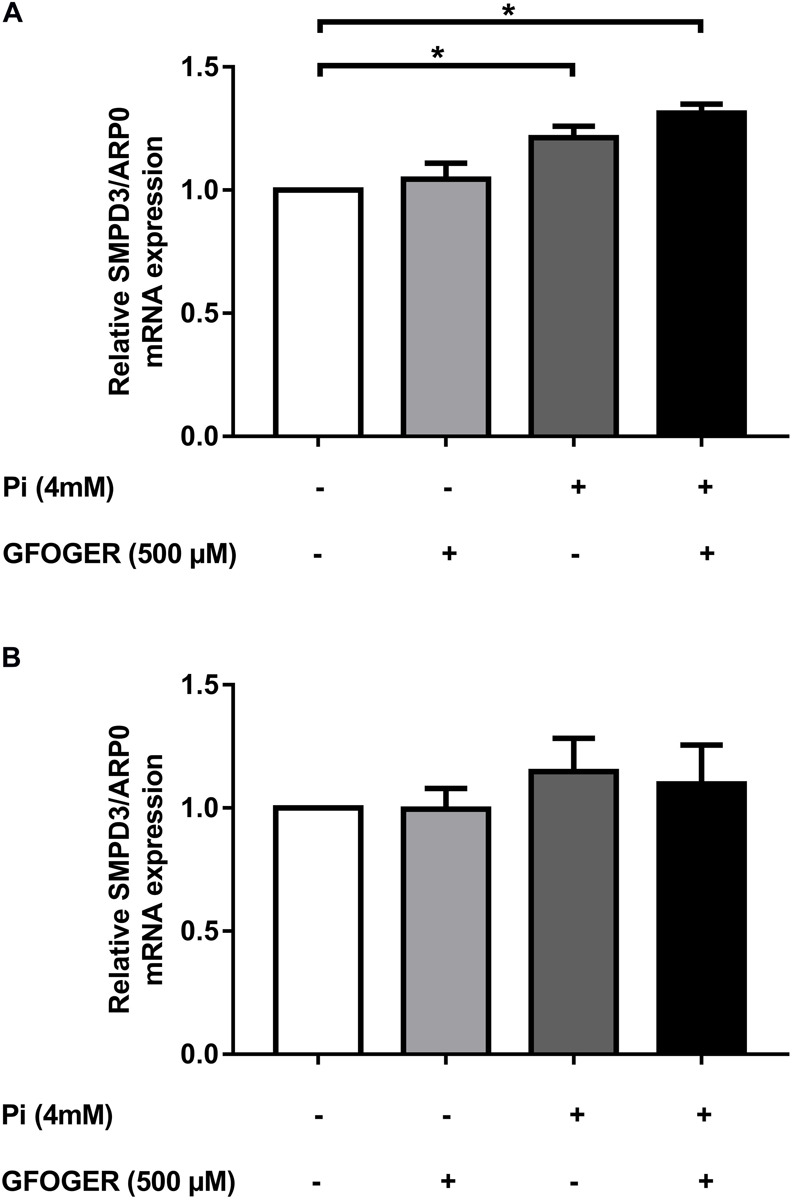
Effects of GFOGER on EVs’ biogenesis. MOVAS-1 cells were incubated with 500 μM GFOGER peptide in the presence or absence of 4 mM Pi for 24 h **(A)** and 8 days **(B)**. Gene expression of EVs’ biogenesis marker (SMPD3) was then quantified by RT-qPCR and normalized to the housekeeping gene ARP0. Data are expressed as mean ± SEM of four independent experiments done in triplicate (*n* = 4). *p < 0.05 vs. 4 mM Pi; Mann–Whitney test.

A hallmark of the calcification process is the upregulated expression of the gene coding for sphinogomyelin phosphodiesterase (SMPD3), an enzyme that mediates the release of EVs into the extracellular matrix (ECM) (Kapustin et al., 2015). In MOVAS-1 cells, as expected, *SMPD3* mRNA expression was greater after 24 h of treatment with 4 mM Pi. Interestingly, this mRNA expression was not only normalized but was even significantly elevated after 24 h of treatment with 500 μM GFOGER peptide. This finding suggested that the presence of GFOGER peptide might not normalize the upregulation of EVs’ biogenesis induced by the calcification process (Figure 3A). After 8 days of treatment, *SMPD3* transcription was normalized under all conditions—indicating that EVs’ biogenesis is no longer affected after several days of Pi and/or GFOGER peptide exposures (Figure 3B).

### GFOGER Peptide Modifies the EVs’ Protein Content

We next used MS-based proteomics to determine variations in the EVs’ protein composition under various conditions. Similar numbers of proteins (about 2,000) were identified in EVs under all conditions, with an FDR of 1% (Supplementary Figure 1A). The enrichment of EVs’ samples under all conditions was also confirmed by the presence of various markers, including tetraspanins (CD9 and CD81), various Rab GTPases (Rab7a, Rab9a, and Rab27b), and components of the endosomal sorting complex required for transport (ESCRT, such as TSG-101, Alix, and flotillin-1) (Supplementary Table 1). A Gene Ontology analysis of cellular component showed that membrane-bound vesicles and EVs contained high levels of EV proteins. In contrast, levels of ribosomal, cytoplasmic, and organelle proteins were low (Supplementary Figure 1B)—confirming the efficiency of our EVs’ extraction protocol. We also detected several proteins involved in VC, such as annexins 2, 5, and 6, ALP, inorganic pyrophosphatase, and metalloproteases (Supplementary Table 1).

**FIGURE 4:**
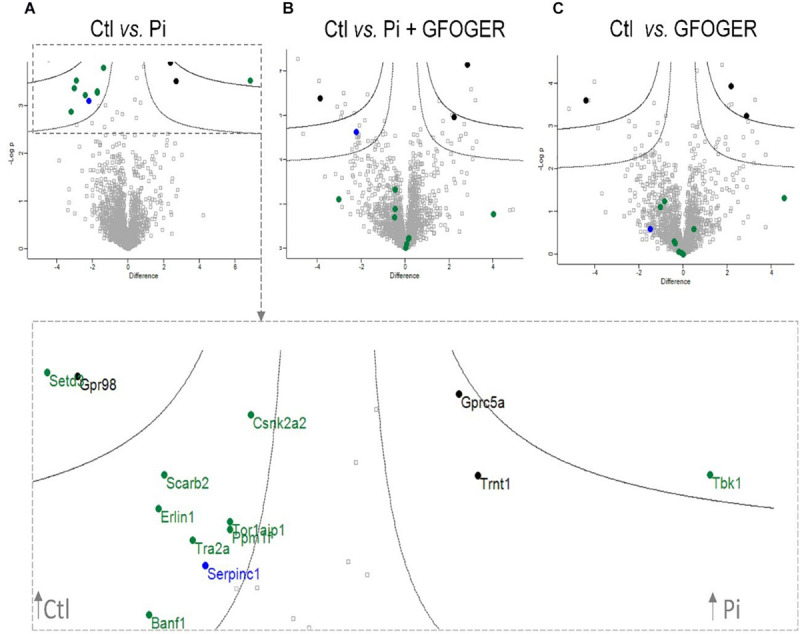
Volcano plots indicating significant GFOGER effects on expression of EVs’ proteins. EVs were prepared from MOVAS-1 cells’ culture media after a 10-day treatment with 4 mM Pi in the presence or absence of 500 μM GFOGER peptide. EVs were prepared from culture media of three independent experiments. Log-transformed *p*-values associated with individual protein against log-transformed fold change in abundance between **(A)** control vs. 4 mM Pi conditions, **(B)** control vs. Pi + GFOGER conditions, and **(C)** control *vs.* GFOGER conditions. Green: proteins altered after Pi and rescued by GFOGER; black: proteins non-specifically altered after Pi; blue: proteins altered after Pi and not rescued by GFOGER.

To investigate the effect of GFOGER peptide on the EVs’ protein cargo, the EVs’ protein profile under different experimental conditions was compared with the control (non-treated) condition. The results of each comparison were represented as volcano plot in which different colors (blue, black, and green) were assigned to sets of proteins modified by the various treatments. All labeled proteins in green were altered upon Pi treatment. Three proteins (in black) were also altered by treatment with GFOGER peptide alone, and so were not directly related to Pi treatment. It is noteworthy that all of the proteins altered by Pi (except antithrombin III, encoded by the *SERPINC1* gene, in blue) were normalized by the addition of GFOGER peptide (in green) (Figure 4).

### GFOGER Peptide Inhibits Osteogenic Marker Expression

**FIGURE 5:**
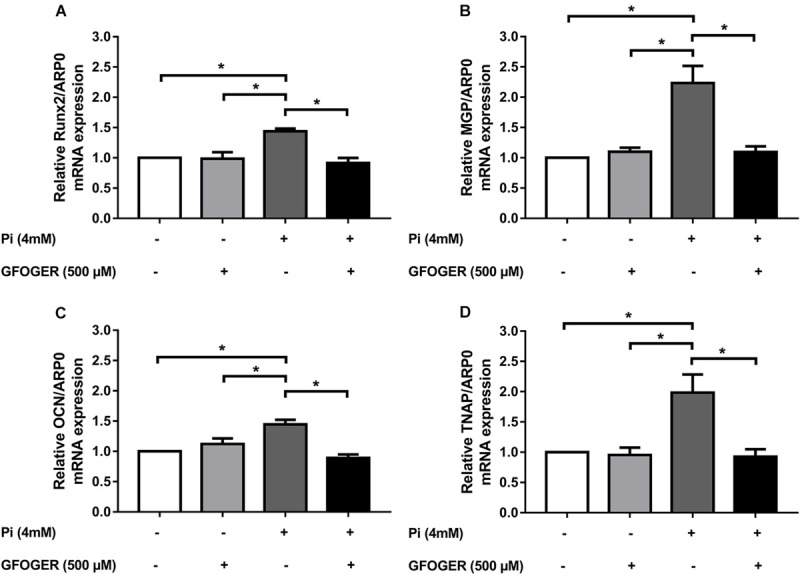
GFOGER peptide decreases the expression of osteogenic markers such as Runx2, MGP, OCN, and TNAP. MOVAS-1 cells were incubated with 500 μM GFOGER peptide in the presence or absence of 4 mM Pi for 8 days. Gene expression of different osteogenic markers was then quantified by RT-qPCR and normalized to the housekeeping gene ARP0. **(A)** Runx2, **(B)** MGP, **(C)** OCN, and **(D)** TNAP. Data are expressed as mean ± SEM of four independent experiments done in triplicate (*n* = 4). *p < 0.05 vs. 4 mM Pi; Mann–Whitney test.

We wanted to investigate whether the GFOGER peptide had any effect on the Pi-induced osteogenic switch of smooth muscle cells. Therefore, we assessed mRNA expression of several osteogenic markers known to play a role in the Pi-induced calcification, i.e., Runx-2, MGP, OCN, and TNAP. The mRNA expression of each osteogenic marker [Runx2, MGP, OCN, and tissue non-specific ALP (TNAP)] was upregulated upon Pi treatment, whereas levels were similar to control values in the presence of Pi and 500 μM GFOGER peptide (^∗^*p* < 0.05 vs. 4 mM Pi; Figure 5); this indicated that GFOGER peptide impairs osteogenic switching in MOVAS-1 cells.

## Discussion

Vascular calcification is an active, multifaceted process with features of bone physiology and metabolism. Like bone formation, VC is characterized by the extensive precipitation of hydroxyapatite crystals within the ECM of blood vessels (Demer and Tintut, 2008; Shanahan Catherine and Crouthamel Matthew, 2011; Durham et al., 2018). VC is also associated with the transformation of VSMCs into osteoblast-like cells. This “osteogenic switching” is characterized by the release of EVs into the ECM and the expression of several bone markers (Steitz Susie and Speer Mei, 2001). EVs are nanoparticles produced under both calcifying and physiological conditions; however, the EVs’ protein content differs according to the condition. For instance, under normal conditions, EVs contain high levels of the calcification inhibitors carboxylated MGP and fetuin-A; under procalcifying conditions, expression of these inhibitors is reduced (Reynolds et al., 2004, 2005; Kapustin et al., 2011; Kapustin and Shanahan, 2016). In a previous work, we showed that VC could be partially inhibited by preventing the interaction between EVs and COL1 (Hodroge et al., 2017). We also showed that a specific six-amino-acid repeat (GFOGER) in COL1 sequence is involved in the interaction with integrins expressed on EVs. In the present study, we hypothesized that synthetic GFOGER peptide would prevent the EV–COL1 interaction and thereby inhibit the calcification process. To test our hypothesis, we studied the GFOGER peptide’s effect on the EVs’ protein content, and calcification and the expression levels of osteogenic markers.

Using the MOVAS-1 cell-based model, we demonstrated that the GFOGER peptide (at 250 and 500 μM) was able to inhibit Pi-induced calcification *in vitro*. We also showed that this inhibition was not due to cytotoxicity because cell proliferation was maintained under all our experimental conditions. The specificity of GFOGER peptide was demonstrated by the use of a modified peptide sequence (GOERFG) which did not inhibit the Pi-induced calcification. Furthermore, we confirmed GFOGER peptide’s inhibitory activity in primary human VSMCs and in an *ex vivo* aortic ring model—both of which mimic VC in the vessel wall *in vivo*. The GFOGER peptide’s inhibitory effect was greater in the aortic ring model (91%) than on isolated cells (58%), suggesting that cells other than VSMCs might be affected by this peptide. Indeed, the aortic ring is a three-dimensional model with a well-structured ECM and that involves many cell types.

As foci for crystal nucleation, calcifying VSMC-derived exosomes are newly recognized key players in the progression of VC (Kapustin et al., 2015; Blaser and Aikawa, 2018). Several researchers have reported on the presence of calcifying EVs in various *in vitro* and *in vivo* models and in the vasculature of patients with CKD or atherosclerotic lesions (Shroff et al., 2010; Kapustin et al., 2011; New Sophie, 2013). Moreover, various studies have indicated that calcification-competent exosomes are enriched by a repertoire of membrane proteins. Along with other proteins that can interfere with and alter the general physiology of recipient cells, these membrane proteins facilitate ECM interaction and mineral nucleation (Kapustin et al., 2011; New Sophie, 2013). Having previously confirmed that direct interaction between EVs and COL1 *via* the GFOGER sequence is a key event in the calcification process, we sought to determine whether or not EV biogenesis and EV protein content were modified in the presence of GFOGER peptide. The first step was to confirm the purity of our isolated EVs’ fraction. Previous proteomic studies had revealed that the quality of EV samples depends on the presence of different markers; this was confirmed by our detection of several markers (such as ESCRT) in an MS-based analysis. In functional terms, the presence of proteins directly associated with calcium ion transport, pyrophosphate degradation, ECM interaction/remodeling, apoptosis, and oxidative stress confirmed that Pi induces the release of EVs equipped with the machinery required for mineralization. For instance, we showed that high-Pi medium modified the expression of proteins involved in several essential processes, such as kinase activity (controlling the cell cycle and inflammatory responses), mRNA binding and histone modification (altering gene expression), phosphatase activity, and calcium ion binding. Our results in this respect are in line with the literature data (Kapustin et al., 2015; Chaudhary et al., 2018).

Furthermore, our proteomic analysis showed that the expression of some Pi-modified EVs’ proteins was normalized upon GFOGER treatment, which therefore appears to counteract the effect of Pi. Strikingly, only one protein (antithrombin III) downregulated by Pi treatment was not normalized by GFOGER peptide. Antithrombin III is a serpin family protease inhibitor involved in the coagulation process. Thrombin exerts effects on vascular cells (including VSMCs) by interacting with members of the protease-activated receptor family. It also has a role in the inflammatory response (via IL-6 and MCP1) and stimulates VSMCs to synthesize collagen and therefore promotes ECM accumulation (Chambers et al., 1998). The link between calcification and coagulation has not yet been clarified. However, previous research has demonstrated that a hypercoagulation state is present in several cardiovascular diseases (Ilcheff et al., 2010). Moreover, a recent study showed that levels of antithrombin III were lower in patients with calcified aortic stenosis than in patients with aortic regurgitation, which suggested the presence of a link between the anticoagulation state and calcification (Mourino-Alvarez et al., 2016). Kapustin et al. also showed that VSMC-derived exosomes simultaneously activate coagulation and induce calcification (Kapustin et al., 2017).

In contrast, most of the normalized proteins in the presence of GFOGER peptide are kinases or phosphatases that control calcification-related intracellular signal pathways through a subtle balance between phosphorylation and dephosphorylation. For example, Ppm1f is a serine/threonine protein phosphatase that regulates multifunctional Ca^++^/calmodulin-dependent protein kinase (CAMKII) (Ishida et al., 2003). It was recently shown that the inhibition of CAMKII is associated with lower calcification in the apolipoprotein E^–/–^ mouse model (Ebenebe et al., 2017). Remarkably, we showed that the level of Ppm1f was low in the presence of Pi; this might lead to the persistent presence of activated, phosphorylated CAMKII and thus an increase of calcification. The addition of GFOGER normalized Ppm1f expression; the likely inhibition of CAMKII might explain (at least in part) the observed lower degree of calcification. Ppm1f and actin-histidine *N*-methyltransferase (Setd3) reportedly interfere with histone de-phosphorylation and methylation, respectively. This type of interference usually changes gene expression and prompts smooth muscle cells to differentiate (Eom et al., 2011).

Our proteomic analysis highlighted another interesting protein: casein kinase II (CKII, encoded by the *Csnk2a2* gene). This highly conserved serine/threonine kinase catalyzes the phosphorylation of proteins involved in the cell cycle and in apoptosis (Singh and Ramji, 2008; Volodina and Shtil, 2012). It is also involved in the phosphorylation of OPN, which inhibits VC (Lasa et al., 1997; Jono et al., 2000; Ohri et al., 2005). Here, we showed that CKII expression was lower in the presence of Pi, suggesting a decline in OPN phosphorylation. However, the presence of GFOGER peptide was able to normalize the expression of CKII, which would probably lead to the phosphorylation of OPN and the inhibition of calcification.

The observed normalization of TANK-binding kinase 1 expression constitutes additional evidence for the GFOGER peptide’s effect on calcification. This serine/threonine protein kinase is involved in the regulation of cell proliferation and apoptosis and in the activation of interferon regulatory factors and NF-κB-mediated signaling pathways; in turn, this triggers inflammation that reportedly favors the osteogenic conversion of VSMCs (Pomerantz and Baltimore, 1999; Voelkl et al., 2018). Indeed, researchers have found that in atherosclerotic lesions, the inflammatory niche constitutes a mixture of cytokines (TNF-α and IL-6) secreted by infiltrated macrophages, and C-reactive proteins, both of which act on VSMCs in a paracrine manner to induce a phenotypic switch and an acceleration of calcification (Tintut et al., 2002; Shao et al., 2010; Voelkl et al., 2019). Here, we showed that Pi exposure was associated with greater TANK-binding kinase 1 expression and thus probably induced inflammation, whereas GFOGER rescued the expression of this protein—suggesting that the peptide inhibits inflammation and thus calcification. The expression levels of several other proteins (see Figure 4) were normalized in the presence of GFOGER peptide, although their relationships with calcification and/or inflammation have not been clearly established. These proteins warrant further investigation.

Interestingly, our proteomic results highlighted a specific role of GFOGER peptide in the normalization of several proteins involved in bone formation indicating that this peptide was able to normalize the expression of various bone-related genes induced by Pi. Our qRT-PCR results confirmed that GFOGER peptide is able to block the osteogenic switching of VSMCs in a direct or indirect manner by normalizing the mRNA expression of Runx2, MGP, OCN, and TNAP. This molecular effect suggests the GFOGER peptide might interact directly with a cell surface receptor that activates signaling pathways and thus modifies gene expression. Another hypothesis could be that GFOGER peptide’s physical–chemical properties and functional groups might interact and complex with Pi ions—thus preventing the latter from entering cells and thus diminishing their effect on gene expression (Itoh et al., 2002; Demer and Tintut, 2008; Farbod et al., 2013). The latter hypothesis seems unlikely, however, because the presence of GFOGER did not counteract Pi-induced upregulation of SMPD3.

Taken as a whole, our results suggest that the GFOGER peptide is a novel and promising therapeutic approach for decreasing VC by (1) inhibiting osteogenic switching of VSMCs and (2) modifying the protein content of VSMC-derived EVs. Our results also open up new research perspectives for assessing the GFOGER peptide’s efficacy in animal and human models.

## Data Availability Statement

Our proteomic dataset has been uploaded to Proteome Xchange repository—accession PXD018169.

## Author Contributions

AM, WD, LY, SD, CG, and VJ contributed to experimentation. CR, PS, SK, CG, AB, and JA contributed to conception and data analysis. AM, AB, and JA contributed to manuscript writing. All authors contributed to the article and approved the submitted version.

## Conflict of Interest

The authors declare that the research was conducted in the absence of any commercial or financial relationships that could be construed as a potential conflict of interest.

## Footnotes

1 www.perseus-framework.org

## References

[B1] Adeney K. L. Siscovick D. S. Ix J. H. Seliger S. L. Shlipak M. G. Jenny N. S. (2009). Association of serum phosphate with vascular and valvular calcification in moderate CKD. *J. Am. Soc. Nephrol. JASN.* 20 381–387. 10.1681/asn.2008040349 19073826 PMC2637048

[B2] Baker M. Robinson S. D. Lechertier T. Barber P. R. Tavora B. D’Amico G. (2011). Use of the mouse aortic ring assay to study angiogenesis. *Nat. Protoc.* 7 89–104. 10.1038/nprot.2011.435 22193302

[B3] Blaser M. C. Aikawa E. (2018). Roles and Regulation of Extracellular Vesicles in Cardiovascular Mineral Metabolism. *Front. Cardiovasc. Med.* 5:6308298. 10.3389/fcvm.2018.00187 30622949 PMC6308298

[B4] Chambers R. C. Dabbagh K. McANULTY R. J. Gray A. J. Blanc-Brude O. P. Laurent G. J. (1998). Thrombin stimulates fibroblast procollagen production via proteolytic activation of protease-activated receptor 1. *Biochem. J.* 333 121–127. 10.1042/bj3330121 9639571 PMC1219564

[B5] Chaudhary S. C. Khalid S. Smethurst V. Monier D. Mobley J. Huet A. (2018). Proteomic profiling of extracellular vesicles released from vascular smooth muscle cells during initiation of phosphate-induced mineralization. *Con. Tissue Res.* 59 55–61. 10.1080/03008207.2018.1444759 29471680 PMC6414064

[B6] Chen N. X. O’Neill K. D. Chen X. Moe S. M. (2008). Annexin-Mediated Matrix Vesicle Calcification in Vascular Smooth Muscle Cells. *J. Bone Miner. Res.* 23 1798–1805. 10.1359/jbmr.080604 18597635 PMC2685487

[B7] Chow B. Rabkin S. W. (2015). The relationship between arterial stiffness and heart failure with preserved ejection fraction: a systemic meta-analysis. *Heart Fail. Rev.* 20 291–303. 10.1007/s10741-015-9471-1 25716909

[B8] Cox J. Mann M. (2008). MaxQuant enables high peptide identification rates, individualized p.p.b.-range mass accuracies and proteome-wide protein quantification. *Nat. Biotechnol.* 26 1367–1372. 10.1038/nbt.1511 19029910

[B9] Demer L. L. Tintut Y. (2008). Vascular calcification: pathobiology of a multifaceted disease. *Circulation* 117 2938–2948. 10.1161/circulationaha.107.743161 18519861 PMC4431628

[B10] Durham A. L. Speer M. Y. Scatena M. Giachelli C. M. Shanahan C. M. (2018). Role of smooth muscle cells in vascular calcification: implications in atherosclerosis and arterial stiffness. *Cardiovasc. Res.* 114 590–600. 10.1093/cvr/cvy010 29514202 PMC5852633

[B11] Ebenebe O. Heather A. Erickson J. (2017). The Role of CaMKII in Atherosclerotic Plaque Calcification in the ApoE-null Mouse. *Heart Lung. Circ.* 26:S65.

[B12] Eom G. H. Kim K.-B. Kim J. H. Kim J.-Y. Kim J.-R. Kee H. J. (2011). Histone methyltransferase SETD3 regulates muscle differentiation. *J. Biol. Chem.* 286 34733–34742. 10.1074/jbc.m110.203307 21832073 PMC3186363

[B13] Farbod K. Nejadnik M. R. Jansen J. A. Leeuwenburgh S. C. G. (2013). Interactions Between Inorganic and Organic Phases in Bone Tissue as a Source of Inspiration for Design of Novel Nanocomposites. *Tissue Eng. B Rev.* 20 173–188. 10.1089/ten.teb.2013.0221 23902258

[B14] Hodroge A. Trécherel E. Cornu M. Darwiche W. Mansouri A. Ait-Mohand K. (2017). Oligogalacturonic acid inhibits vascular calcification by two mechanisms. *Arterioscler. Thromb. Vasc. Biol.* 37, 1391–1401. 10.1161/atvbaha.117.309513 28522698

[B15] Ilcheff B. J. Sylvia H. Evren K. Peter K. Van Oerle R. Kristien W. (2010). Early Atherosclerosis Exhibits an Enhanced Procoagulant State. *Circulation* 122 821–830. 10.1161/circulationaha.109.907121 20697022

[B16] Ishida A. Shigeri Y. Taniguchi T. Kameshita I. (2003). Protein phosphatases that regulate multifunctional Ca2+/calmodulin-dependent protein kinases: from biochemistry to pharmacology. *Pharmacol. Ther.* 100 291–305. 10.1016/j.pharmthera.2003.09.003 14652114

[B17] Issa H. Hénaut L. Abdallah J. B. Boudot C. Lenglet G. Avondo C. (2019). Activation of the calcium-sensing receptor in human valvular interstitial cells promotes calcification. *J. Mol. Cell Cardiol.* 129 2–12. 10.1016/j.yjmcc.2019.01.021 30769016

[B18] Itoh D. Yoneda S. Kuroda S. Kondo H. Umezawa A. Ohya K. (2002). Enhancement of osteogenesis on hydroxyapatite surface coated with synthetic peptide (EEEEEEEPRGDT) in vitro. *J. Biomed. Mater Res.* 62 292–298. 10.1002/jbm.10338 12209950

[B19] Jono S. Peinado C. Giachelli C. M. (2000). Phosphorylation of osteopontin is required for inhibition of vascular smooth muscle cell calcification. *J. Biol. Chem.* 275 20197–20203. 10.1074/jbc.m909174199 10766759

[B20] Kapustin A. N. Michael S. Schurgers L. J. Reynolds J. L. Rosamund M. Alexander H. (2017). Prothrombin Loading of Vascular Smooth Muscle Cell–Derived Exosomes Regulates Coagulation and Calcification. *Arterioscler. Thromb. Vasc. Biol.* 37 e22–e32. 28104608 10.1161/ATVBAHA.116.308886

[B21] Kapustin A. N. Shanahan C. M. (2016). Emerging roles for vascular smooth muscle cell exosomes in calcification and coagulation. *J. Physiol.* 594 2905–2914. 10.1113/jp271340 26864864 PMC4887700

[B22] Kapustin A. N. Chatrou M. L. L. Drozdov I. Zheng Y. Davidson S. M. Soong D. (2015). Vascular smooth muscle cell calcification is mediated by regulated exosome secretion. *Circ. Res.* 116 1312–1323. 10.1161/circresaha.116.305012 25711438

[B23] Kapustin A. N. Davies J. D. Reynolds J. L. McNair R. Jones G. T. Sidibe A. (2011). Calcium regulates key components of vascular smooth muscle cell-derived matrix vesicles to enhance mineralization. *Circ. Res.* 109 e1–e12. 21566214 10.1161/CIRCRESAHA.110.238808

[B24] Lasa M. Chang P. L. Prince C. W. Pinna L. A. (1997). Phosphorylation of osteopontin by Golgi apparatus casein kinase. *Biochem. Biophys. Res. Commun.* 240 602–605. 10.1006/bbrc.1997.7702 9398611

[B25] Luber C. A. Cox J. Lauterbach H. Fancke B. Selbach M. Tschopp J. (2010). Quantitative Proteomics Reveals Subset-Specific Viral Recognition in Dendritic Cells. *Immunity* 32 279–289. 10.1016/j.immuni.2010.01.013 20171123

[B26] Mourino-Alvarez L. Baldan-Martin M. Gonzalez-Calero L. Martinez-Laborde C. Sastre-Oliva T. Moreno-Luna R. (2016). Patients with calcific aortic stenosis exhibit systemic molecular evidence of ischemia, enhanced coagulation, oxidative stress and impaired cholesterol transport. *Int. J. Cardiol.* 225 99–106. 10.1016/j.ijcard.2016.09.089 27716559

[B27] New Sophie E. P. (2013). Aikawa Elena. Role of Extracellular Vesicles in De Novo Mineralization. *Arterioscler. Thromb. Vasc. Biol.* 33 1753–1758. 10.1161/atvbaha.112.300128 23766262 PMC3788633

[B28] Nicoll R. Henein M. Y. (2014). The predictive value of arterial and valvular calcification for mortality and cardiovascular events. *IJC Heart Vessels* 3 1–5. 10.1016/j.ijchv.2014.02.001 29450162 PMC5801264

[B29] Ohri R. Tung E. Rajachar R. Giachelli C. M. (2005). Mitigation of Ectopic Calcification in Osteopontin-Deficient Mice by Exogenous Osteopontin. *Calcif. Tissue Int.* 76 307–315. 10.1007/s00223-004-0071-7 15812576

[B30] Pêcher J. Pires V. Djaafri I. Da Nascimento S. Fauvel-Lafève F. Legrand C. (2009). Circular dichroism studies of type III collagen mimetic peptides with anti- or pro-aggregant activities on human platelets. *Eur. J. Med. Chem.* 44 2643–2650. 10.1016/j.ejmech.2008.10.026 19056149

[B31] Perez-Riverol Y. Csordas A. Bai J. Bernal-Llinares M. Hewapathirana S. Kundu D. J. (2019). The PRIDE database and related tools and resources in 2019: improving support for quantification data. *Nucleic Acids Res.* 47 D442–D450. 30395289 10.1093/nar/gky1106 PMC6323896

[B32] Peterson G. L. (1977). . A simplification of the protein assay method of Lowry et al. which is more generally applicable. *Anal. Biochem.* 83 346–356. 10.1016/0003-2697(77)90043-4 603028

[B33] Pomerantz J. L. Baltimore D. (1999). NF-kappaB activation by a signaling complex containing TRAF2, TANK and TBK1, a novel IKK-related kinase. *EMBO J.* 18 6694–6704. 10.1093/emboj/18.23.6694 10581243 PMC1171732

[B34] Reynolds J. L. Joannides A. J. Skepper J. N. McNair R. Schurgers L. J. Proudfoot D. (2004). Human vascular smooth muscle cells undergo vesicle-mediated calcification in response to changes in extracellular calcium and phosphate concentrations: a potential mechanism for accelerated vascular calcification in ESRD. *J. Am. Soc. Nephrol. JASN.* 15 2857–2867. 10.1097/01.asn.0000141960.01035.28 15504939

[B35] Reynolds J. L. Skepper J. N. McNair R. Kasama T. Gupta K. Weissberg P. L. (2005). Multifunctional Roles for Serum Protein Fetuin-A in Inhibition of Human Vascular Smooth Muscle Cell Calcification. *J. Am. Soc. Nephrol.* 16 2920–2930. 10.1681/asn.2004100895 16093453

[B36] Russo D. Morrone L. F. Errichiello C. De Gregorio M. G. Imbriaco M. Battaglia Y. (2014). Impact of BMI on cardiovascular events, renal function, and coronary artery calcification. *Blood Purif.* 38 1–6. 10.1159/000362862 25196674

[B37] Sarkar B. C. Chauhan U. P. (1967). A new method for determining micro quantities of calcium in biological materials. *Anal. Biochem.* 20 155–166. 10.1016/0003-2697(67)90273-4 6071917

[B38] Schlieper G. Schurgers L. Brandenburg V. Reutelingsperger C. Floege J. (2016). Vascular calcification in chronic kidney disease: an update. *Nephrol. Dial Transplant.* 31 31–39. 25916871 10.1093/ndt/gfv111

[B39] Shanahan Catherine M. Crouthamel Matthew H. (2011). Kapustin Alexander, Giachelli Cecilia M., Towler Dwight A. Arterial Calcification in Chronic Kidney Disease: Key Roles for Calcium and Phosphate. *Circ. Res.* 109 697–711. 10.1161/circresaha.110.234914 21885837 PMC3249146

[B40] Shao J.-S. Cheng S.-L. Sadhu J. Towler D. A. (2010). Inflammation and the Osteogenic Regulation of Vascular Calcification: A Review & Perspective. *Hypertension* 55 579–592. 10.1161/hypertensionaha.109.134205 20101002 PMC2853014

[B41] Shroff R. C. McNair R. Skepper J. N. Figg N. Schurgers L. J. Deanfield J. (2010). Chronic Mineral Dysregulation Promotes Vascular Smooth Muscle Cell Adaptation and Extracellular Matrix Calcification. *J. Am. Soc. Nephrol.* 21 103–112. 10.1681/asn.2009060640 19959717 PMC2799273

[B42] Singh N. N. Ramji D. P. (2008). Protein kinase CK2, an important regulator of the inflammatory response? *J. Mol. Med.* 86:887. 10.1007/s00109-008-0352-0 18437331

[B43] Speer Mei Y. Hsueh-Ying Y. Thea B. Elizabeth L. Amy L. Wei-Ling L. (2009). Smooth Muscle Cells Give Rise to Osteochondrogenic Precursors and Chondrocytes in Calcifying Arteries. *Circ. Res.* 104 733–741. 10.1161/circresaha.108.183053 19197075 PMC2716055

[B44] Steitz Susie A. Speer Mei Y. (2001). Curinga Gabrielle, Yang Hsueh-Ying, Haynes Paul, Aebersold Ruedi, et al. Smooth Muscle Cell Phenotypic Transition Associated With Calcification. *Circ. Res.* 89 1147–1154. 10.1161/hh2401.101070 11739279

[B45] Théry C. Amigorena S. Raposo G. Clayton A. (2006). Isolation and characterization of exosomes from cell culture supernatants and biological fluids. *Curr. Protoc. Cell Biol.* 3:3.22. 10.1002/0471143030.cb0322s30 18228490

[B46] Thompson B. Towler D. A. (2012). Arterial calcification and bone physiology: role of the bone–vascular axis. *Nat. Rev. Endocrinol.* 8 529–543. 10.1038/nrendo.2012.36 22473330 PMC3423589

[B47] Tintut Y. Patel J. Territo M. Saini T. Parhami F. Demer L. L. (2002). Monocyte/macrophage regulation of vascular calcification in vitro. *Circulation* 105 650–655. 10.1161/hc0502.102969 11827934

[B48] Voelkl J. Lang F. Eckardt K.-U. Amann K. Kuro-o M. Pasch A. (2019). Signaling pathways involved in vascular smooth muscle cell calcification during hyperphosphatemia. *Cell Mol. Life Sci.* 76 2077–2091. 30887097 10.1007/s00018-019-03054-z PMC6502780

[B49] Voelkl J. Luong T. T. D. Tuffaha R. Musculus K. Auer T. Lian X. (2018). SGK1 induces vascular smooth muscle cell calcification through NF-κ B signaling. *J. Clin. Invest.* 128 3024–3040. 10.1172/jci96477 29889103 PMC6025998

[B50] Volodina Y. L. Shtil A. A. (2012). Casein kinase 2, a versatile regulator of cell survival. *Mol. Biol.* 46 381–390. 10.1134/s0026893312020203 22888632

[B51] Wu M. Rementer C. Giachelli C. M. (2013). Vascular calcification: an update on mechanisms and challenges in treatment. *Calcif. Tissue Int.* 93 365–373. 10.1007/s00223-013-9712-z 23456027 PMC3714357

[B52] Xiao G. Jiang D. Ge C. Zhao Z. Lai Y. Boules H. (2005). Cooperative Interactions between Activating Transcription Factor 4 and Runx2/Cbfa1 Stimulate Osteoblast-specific Osteocalcin Gene Expression. *J. Biol. Chem.* 280 30689–30696. 10.1074/jbc.m500750200 16000305

