# Supplementary material for: GFOGER Peptide Modifies the Protein Content of Extracellular Vesicles and Inhibits Vascular Calcification
Source: Front Cell Dev Biol. 2020 Nov 30;8:589761. doi: 10.3389/fcell.2020.589761 (PMC7734313; doi:10.3389/fcell.2020.589761)
Supplement: Supplementary file 1 [file Data_Sheet_1.docx]

**Supplemental Table I**

| **EVs Proteins Families** | **Detected Proteins** |
| --- | --- |
| **Tetraspanins** | CD9, CD81 |
| **ESCRT components** | Alix**,** Tsg101, Vps4, Vps 28, Flotillin 1,2 and Syntenin-1 |
| **Rab GTPases** | Rab 7a, Rab 9a, Rab 27b, Rab 35, Rab 5a, 5b, 5c. |
| **Different Proteins associated with EVs pathophysiology** | - Integrins α1, ß1 and ß5 - Annexins 2,5 and 6 - Alkaline Phosphatase (ALP) - Inorganic Pyrophosphatase (ANK) - Kinases (PKC, Tyrosine Kinase and MAPK) - Metalloproteases (MMP9 and MMP 10) - Vascular Cell Adhesion Molecule (VCAM-1) - Scramblases 1 and 3 - Phospholipases D3 - Glutathione-S-Transferase - Apoptosis inducing factors (BAX1 and BAX2) - Cytokine receptor 3 |

Detection of different exosomal markers and proteins related to EVs pathophysiology.
