# Supplementary material for: GFOGER Peptide Modifies the Protein Content of Extracellular Vesicles and Inhibits Vascular Calcification
Source: Front Cell Dev Biol. 2020 Nov 30;8:589761. doi: 10.3389/fcell.2020.589761 (PMC7734313; doi:10.3389/fcell.2020.589761)
Supplement: Supplementary file 2 [file Data_Sheet_2.docx]

**Supplemental Figure 1**


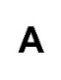


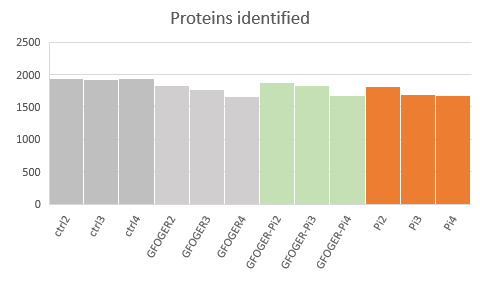


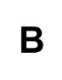


**Figure I. Quantitative and Qualitative overview of EVs proteome.** EVs were prepared form MOVAS-1 cell culture media after 10 days of treatment with different experimental conditions**. (A)** Comparable protein quantities of the different samples. **(B)** Gene Ontology (GO) enrichments of EVs proteome based on cellular localization. EVs samples were prepared from culture media of three independent experiments (n=3).
