# Supplementary figures and images for: GFOGER Peptide Modifies the Protein Content of Extracellular Vesicles and Inhibits Vascular Calcification
Source: Front Cell Dev Biol. 2020 Nov 30;8:589761. doi: 10.3389/fcell.2020.589761 (PMC7734313; doi:10.3389/fcell.2020.589761)

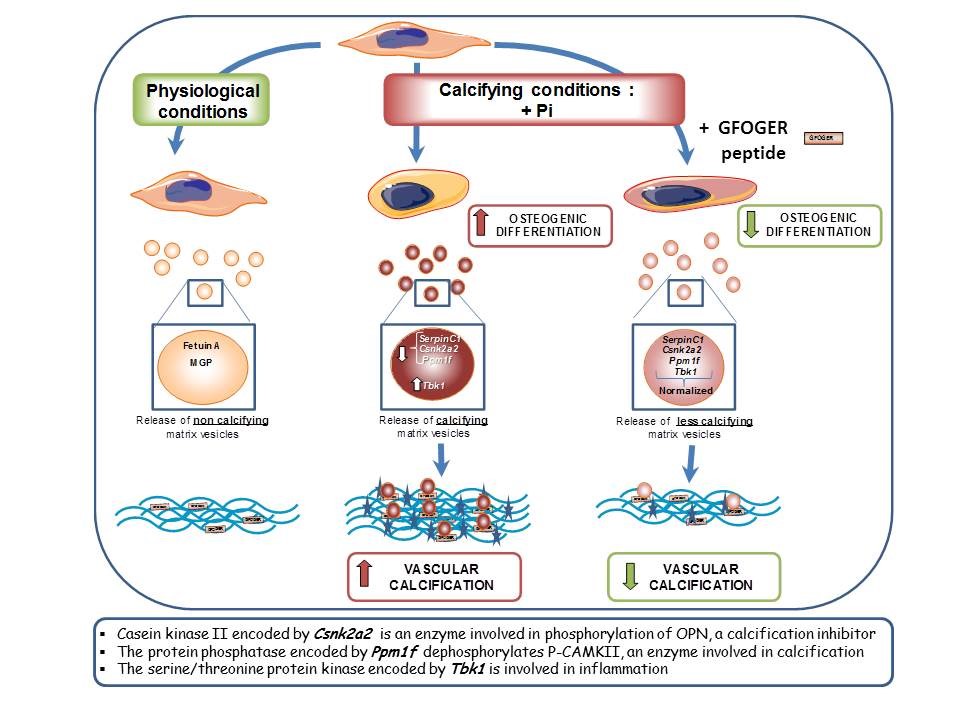

Supplement: Supplementary file 3 [file Image_1.JPEG]
